# Clinical impact of body mass index on bactibilia and bacteremia

**DOI:** 10.1186/1471-230X-14-104

**Published:** 2014-06-05

**Authors:** Chang Seok Bang, Jai Hoon Yoon, Youn Jeong Kim, Jin Bong Kim, Gwang Ho Baik, Ki Tae Suk, Yeon Soo Kim, Dong Joon Kim

**Affiliations:** 1 Department of Internal Medicine, Hallym University College of Medicine, Chuncheon, South Korea; 2 Department of Internal Medicine, The Catholic University of Korea College of Medicine, Seoul, South Korea

**Keywords:** Bacteremia, Cholecystitis, Obesity

## Abstract

**Background:**

The aim of this study was to evaluate the association between obesity and infected bile or bacteremia in patients with acute calculous cholecystitis.

**Methods:**

Authors analyzed the medical records of 139 patients who had undergone cholecystectomy for the treatment of acute calculous cholecystitis from January 2007 to June 2013 in a single teaching hospital. Association of body mass index (BMI) with bactibilia and bacteremia was assessed using univariate and multivariate analysis. Clinical findings and biliary infection related data were recorded for the following variables: gender, age, alcohol and smoking history, the results of blood and bile cultures, cholesterolosis, diabetes, hypertension, and duration of the hospital stay.

**Results:**

The microbial culture rate of bactibilia and bacteremia were 50.4% and 21.6%, respectively. In the univariate analysis, bacteremia was associated with bactibilia (OR: 4.33, *p* = *0.002*). In the multivariate analysis for the risk factors of bactibilia, BMI and bacteremia were related with bactibilia (OR: 0.59, 95% CI: 0.42-0.84, *p = 0.003*) (OR: 3.32, 95% CI: 1.22-9, *p = 0.02*). In the multivariate analysis for the risk factors of bacteremia, BMI, bactibilia and age were related with bacteremia (OR: 0.76, 95% CI: 0.59-0.99, *p = 0.04*) (OR: 3.46, 95% CI: 1.27-9.45, *p = 0.02*) (OR: 1.05, 95% CI: 1.01-1.09, *p = 0.02*).

**Conclusion:**

In this retrospective study, BMI was inversely correlated with bacteremia or bactibilia, which means obese or overweight patients are less likely to be associated with bacteremia or bactibilia in patients with acute calculous cholecystitis.

## Background

Obesity is a well-established risk factor for cholesterol gallstone and subsequent cholecystectomy [1-4]. This is because of the increased cholesterol synthesis or secretion associated with glucose intolerance and insulin resistance [2,5]. Gallbladder (GB) hypomotility secondary to obesity or autonomic neuropathy has also been proposed as one of the mechanisms [6,7]. However, the association between obesity and bactibilia or bacteremia, which have been postulated as one of the mechanisms or manifestations of cholecystitis remains unclear. Selecting empirical antibiotics in patients with bactibilia or bacteremia is also important.

The present study aims to provide an association between obesity and bactibilia or bacteremia in patients with acute calculous cholecystitis and to obtain isolated bacterial profiles of bile and blood to help direct empirical therapy.

## Methods

### Ethics statement

This study was conducted according to the principles expressed in the Declaration of Helsinki and approved by institutional review board of Chuncheon Sacred Heart hospital before initiating study (2013–84). Patient records or information was anonymized and de-identified prior to analysis.

### Patients and methods

**Figure 1:**
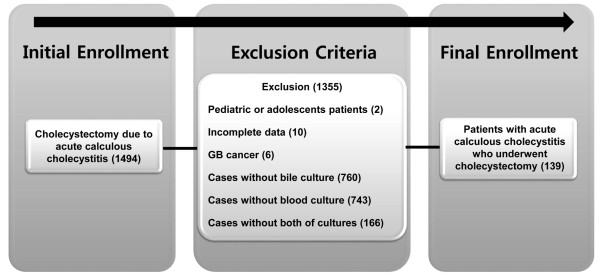
Study flow diagram.

Between January 2007 and June 2013, 1494 cholecystectomy cases due to acute calculous cholecystitis were initially detected in a single teaching hospital of Korea. Pediatric or adolescent patients (age under 18), incomplete data, patients with GB cancer and patients without laboratory results of bile or blood culture were all excluded from this study. The exact distribution of excluded cases are as follows: pediatric or adolescent patients (2), incomplete data (10), GB cancer (6), cases without laboratory results of bile culture (760), blood culture (743), and both of cultures (166) (Figure 1).

A total of 139 eligible patients who had undergone cholecystectomy for the treatment of acute calculous cholecystitis were retrospectively investigated. The association between body mass index (BMI) with bactibilia and bacteremia was assessed using univariate and multivariate analysis. Clinical findings and biliary infection related data were recorded for the following variables: gender, age, alcohol and smoking history, the results of blood and bile cultures, cholesterolosis, diabetes (DM), hypertension (HTN), and duration of the hospital stay. BMI (kg/m^2^) was calculated as the patient’s body weight divided by the square of the height. The BMI was categorized and analyzed according to the recommendation of World Health Organization Expert Consultation (Asian population standard): stage 1 (<23), stage 2 (23 ≤ BMI < 25), and stage 3 (25 ≤ BMI) [8].

Blood culture was performed to identify the responsible microorganism for the infection before initiating antibiotics (BACTEC 9240 system). A minimum of 10 mL of blood was taken separately from more than two different peripheral veins after cleansing the puncture site and the top of the culture bottles with alcohol or povidone-iodine sterilization. Bile culture was performed using accessible specimen through percutaneous transhepatic gallbladder drainage tube or endoscopic nasobiliary drainage catheter after cleansing the tips of the tube or catheter and the top of the culture bottles. All the bacteriologic isolation and detection of the antimicrobial resistance procedure were performed by the in-hospital laboratory medicine department. Microbial culture rate from blood or bile and antimicrobial resistance pattern was recorded and analyzed.

### Statistical analysis

The continuous variables which showed normal distribution were expressed using the mean and standard deviations (SD) and which did not show normal distribution using the median and interquartile ranges. Student’s *t* test and Mann–Whitney test were used to evaluate the continuous variables. Fisher’s exact test and linear-by-linear association test were used to assess the categorical variables. A multivariate logistic regression test was used to detect the independent risk factors related to bactibilia or bacteremia. A *p* value *< 0.05* was considered significant for all tests. All the analysis were performed using the SPSS software, version 18.0 (SPSS Inc., Chicago, IL, USA).

## Results

### Characteristic of patients

Among the 139 eligible patients, 70 patients (50.4%) showed bactibilia and 30 patients (21.6%) showed bacteremia. The median age was 68 (interquartile range: 58–75) in the total enrolled population: 69.5 (58–75) in patients with bactiblia, and 73 (61.75-81) in patients with bacteremia. Male predominance (59%) was observed in the total number of the patients: patients with bactibilia (60%) and with bacteremia (56.7%). The mean BMI (±SD) was 24 ± 3.4 in the total patients: 23.7 ± 3.8 in patients with bactibilia, and 23.7 ± 4.2 with bacteremia. The distribution of BMI stage in the total number of the patients is as follows: stage 1 (41.7%), stage 2 (19.4%), and stage 3 (38.9%). The clinical characteristics of the total enrolled patients are summarized in Table 1. The clinical findings of the patients with bactibilia and bacteremia are shown in Tables 2 and 3, respectively.

**Table 1:** Clinical characteristics of total patients

**Characteristics**	**Total (*n*** **= 139)**
Age (years), Median (Interquartile range)	68 (58–75)
Sex, number (%)	
Male	82 (59%)
Female	57 (41%)
Smoking (%)	
Number of patients	20 (14.4%)
Alcohol (%)	
Number of patients	36 (25.9%)
BMI, Mean ± SD	24 ± 3.4
BMI stage (%)	
Stage 1 (<23)	58 (41.7%)
Stage 2 (23 ≤ BMI < 25)	27 (19.4%)
Stage 3 (25 ≤ BMI)	54 (38.9%)
Cholesterolosis (%)	12 (8.6%)
Duration of hospital stay	21.5 ± 15.8
Mean ± SD (days)	
HTN (%)	60 (43.2%)
DM (%)	23 (16.5%)

BMI: Body mass index; SD: Standard deviation; HTN: Hypertension; DM: Diabetes mellitus.

**Table 2:** Univariate analysis for risk factors of bactibilia

**Characteristics**	**Bactibilia**	**No bactibilia**	***p***
	**(*n*** **= 70, 50.4%)**	**(*n*** **= 69, 49.6%)**	
Age (years), Median (Interquartile range)	69.5 (58–75)	67 (57–76)	*0.67*
Sex, number (%)			*0.86*
Male	42 (60%)	40 (58%)	
Female	28 (40%)	29 (42%)	
Smoking (%)			*0.23*
Number of patients	13 (18.6%)	7 (10.1%)	
Alcohol (%)			*0.18*
Number of patients	22 (31.4%)	14 (20.3%)	
BMI, Mean ± SD	23.7 ± 3.8	24.2 ± 3.1	*0.39*
BMI stage (%)			*> 0.99*
Stage 1 (<23)	29 (41.4%)	29 (42%)	
Stage 2 (23 ≤ BMI < 25)	14 (20%)	13 (18.8%)	
Stage 3 (25 ≤ BMI)	27 (38.6%)	27 (39.1%)	
Cholesterolosis (%)	4 (5.7%)	8 (11.6%)	*0.24*
Duration of hospital stay Mean ± SD (days)	22.4 ± 17	20.5 ± 14.4	*0.49*
HTN (%)	27 (38.6%)	33 (47.8%)	*0.31*
DM (%)	13 (18.6%)	10 (14.5%)	*0.65*

BMI: Body mass index; SD: Standard deviation; HTN: Hypertension; DM: Diabetes mellitus.

**Table 3:** Univariate analysis for risk factors of bacteremia

**Characteristics**	**Bacteremia**	**No bacteremia**	***p***
	**(*n*** **= 30, 21.6%)**	**(*n*** **= 109, 78.4%)**	
Age (years), Median (Interquartile range)	73 (61.75–81)	67 (57.5–74)	*0.02*
Sex, number (%)			*0.84*
Male	17 (56.7%)	65 (59.6%)	
Female	13 (43.3%)	44 (40.4%)	
Smoking (%)			*0.38*
Number of patients	6 (20%)	14 (12.8%)	
Alcohol (%)			*0.64*
Number of patients	9 (30%)	27 (24.8%)	
BMI, Mean ± SD	23.7 ± 4.2	24 ± 3.2	*0.70*
BMI stage (%)			*0.84*
Stage 1 (<23)	14 (46.7%)	44 (40.4%)	
Stage 2 (23 ≤ BMI < 25)	2 (6.7%)	25 (22.9%)	
Stage 3 (25 ≤ BMI)	14 (46.7%)	40 (36.7%)	
Cholesterolosis (%)	3 (10%)	9 (8.3%)	*0.72*
Duration of hospital stay Mean ± SD (days)	20.8 ± 13.9	21.6 ± 16.3	*0.80*
HTN (%)	15 (50%)	45 (41.3%)	*0.41*
DM (%)	2 (6.7%)	21 (19.3%)	*0.16*

BMI: Body mass index; SD: Standard deviation; HTN: Hypertension; DM: Diabetes mellitus.

### Univariate analysis for bactibilia and bacteremia

In the univariate analysis, bacteremia was associated with bactibilia (OR: 4.33, *p = 0.002*). In patients with both of bactibilia and bacteremia, 50% (11/22) showed concordant cultured organism. However, BMI was not associated with bacteremia or bactibilia, although there were inverse relation trends [(bactibilia *vs.* no bactibilia; 23.7 ± 3.8 *vs.* 24.2 ± 3.1, *p = 0.39*), (bacteremia *vs.* no bacteremia; 23.7 ± 4.2 *vs.* 24 ± 3.2, *p = 0.70*)]. BMI stage also showed associations with neither the patients with bactibilia, nor the patients with bacteremia (*p > 0.99* and *p = 0.84*). Only the age was different between the patients with bacteremia and the patients without bacteremia [median (interquartile ranges), 73 (61.75-81) *vs.* 67 (57.5-74), *p = 0.02*] (Tables 2 and 3).

### Multivariate analysis for bactibilia and bacteremia

In the multivariate analysis for the risk factors of bactibilia, BMI and bacteremia were related with bactibilia (OR: 0.59, 95% CI: 0.42-0.84, *p = 0.003*) (OR: 3.32, 95% CI: 1.22-9, *p = 0.02*). In the multivariate analysis for the risk factors of bacteremia, BMI, bactibilia and age were related with bacteremia (OR: 0.76, 95% CI: 0.59-0.99, *p = 0.04*) (OR: 3.46, 95% CI: 1.27-9.45, *p = 0.02*), (OR: 1.05, 95% CI: 1.01-1.09, *p = 0.02*) (Table 4).

**Table 4:** Multivariate logistic regression analysis for the risk factors of bactibilia and bacteremia

**Variables for bactibilia**	**OR (95% CI)**	***p***	**Variables for bactemia**	**OR (95% CI)**	***p***
BMI	0.59 (0.42–0.84)	*0.003*	BMI	0.76 (0.59–0.99)	*0.04*
Bacteremia	3.32 (1.22–9)	*0.02*	Bactibilia	3.46 (1.27–9.45)	*0.02*
Age	NS	*NS*	Age	1.05 (1.01–1.09)	*0.02*

BMI: Body mass index; NS: Statistically non-significant; OR: Odds ratio; CI: Confidence interval.

### Bacteriologic analysis

The microbial culture rate of biliary bacteria and bacteremia were 50.4% and 21.6%, respectively. The most common pathogen of bile and blood culture was commonly *Escherichia coli* (*E. coli*) [(*n* = 22, 31.4%), (*n* = 12, 40%)], followed by *Klebsiella pneumonia* (*n* = 19, 27.1%) and *Pseudomonas aeruginosa* (*n* = 11, 15.7%) in the bile samples, and *Klebsiella pneumonia* (*n* = 4, 13.3%), *Pseudomonas aeruginosa* (*n* = 4, 13.3%), and *Staphylococcus spp.* (*n* = 6, 20%) in the blood samples (Table 5). Antimicrobial resistance rate of *E. coli* was 59.1% of bile specimens and 16.7% of blood specimens. The proportion of extended-spectrum beta-lactamase (ESBL) producing organism was estimated as 27.3% of *E. coli* in bile specimens, but it was not detected in the blood specimens (Table 6). Among the *Klebsiella pneumonia* species (spp.), 52.9% showed ESBL producing organism in bile specimens and 50% showed in blood specimens. Vancomycin-resistant enterococci (VRE) was detected only in *Enterococcus casseliflavus* spp. as 25%. One *Acinetobacter baumannii* was isolated from the bile culture and was identified as imipenem-resistant *Acinetobacter baumannii* (IRAB). One *Staphylococcus epidermidis* was isolated from the blood culture and was identified as Methicillin-resistant coagulase-negative *Staphylococci* (MR-CNS) (Table 6).

**Table 5:** Total isolated organisms in bile and blood culture

**Organism**	**Bactibilia**	**Bacteremia**
	**(*n*** **= 70, 50.4%)**	**(*n*** **= 30, 21.6%)**
*E. coli*	22 (31.4%)	12 (40%)
*Klebsiella* spp.	19 (27.1%)	4 (13.3%)
*Klebsiella pneumonia*	17 (24.3%)	4 (13.3%)
*Klebsiella ozaenae*	1 (1.4%)	
*Klebsiella oxytoca*	1 (1.4%)	
*Enterococcus* spp.	14 (20%)	2 (6.7%)
*Enterococcus faecium*	6 (8.6%)	2 (6.7%)
*Enterococcus casseliflavus*	4 (5.7%)	
*Enterococcus faecalis*	3 (4.3%)	
*Enterococcus durans*	1 (1.4%)	
*Pseudomonas aeruginosa*	11 (15.7%)	4 (13.3%)
*Enterobacter cloacae*	4 (5.7%)	
*Aeromonas* spp.	2 (2.9%)	1 (3.3%)
*Aeromonas hydrophilia*	1 (1.4%)	
*Aeromonas veronii biovar sobria*	1 (1.4%)	
*Stenotrophomonas maltophilia*	2 (2.9%)	·
*Raoultella planticola*	1 (1.4%)	·
*Acinetobacter baumannii*	1 (1.4%)	·
*Staphylococcus* spp.		6 (20%)
*Staphylococcus homonis*		4 (13.3%)
*Staphylococcus epidermidis*		1 (3.3%)
*Staphylococcus auricularis*		1 (3.3%)
*Streptococcus viridans*	·	1 (3.3%)
*Morganella morganii*	·	1 (3.3%)

spp.: species.

**Table 6:** Antimicrobial resistance rate of isolated organisms

**Organism**	**Bactibilia (*n*** **= 70, 50.4%)**	**Bacteremia (*n*** **= 30, 21.6%)**
*E. coli*	13/22 (59.1%) ESBL: 6/22 (27.3%)	2/12 (16.7%) ESBL: 0
*Klebsiella* spp.	19/19 (100%) ESBL 17/19 (89.5%)	3/4 (75%) ESBL 2/4 (50%)
*Klebsiella pneumonia*	17/17 (100%) ESBL 9/17 (52.9%)	3/4 (75%) ESBL 2/4 (50%)
*Klebsiella ozaenae*	1/1 (100%)	
*Klebsiella oxytoca*	1/1 (100%)	
*Enterococcus* spp.	13/14 (92.9%)	2/2 (100%)
*Enterococcus faecium*	6/6 (100%)	2/2 (100%)
*Enterococcus casseliflavus*	4/4 (100%) VRE 1/4 (25%)	
*Enterococcus faecalis*	3/3 (100%)	
*Enterococcus durans*	0/1 (0%)	
*Pseudomonas aeruginosa*	10/11 (90.9%)	4/4 (100%)
*Enterobacter cloacae*	4/4 (100%)	
*Aeromonas* spp.	2/2 (100%)	1/1 (100%)
*Aeromonas hydrophilia*	1/1 (100%)	
*Aeromonas veronii biovar sobria*	1/1 (100%)	
*Stenotrophomonas maltophilia*	1/2 (50%)	·
*Raoultella planticola*	1/1 (100%)	·
*Acinetobacter baumannii*	1/1 (100%) IRAB 1/1 (100%)	·
*Staphylococcus* spp.	·	6/6 (100%)
*Staphylococcus homonis*		4/4 (100%)
*Staphylococcus epidermidis*		1/1 (100%) MR-CNS 1/1 (100%)
*Staphylococcus auricularis*		1/1 (100%)
*Streptococcus viridans*	·	1/1 (100%)
*Morganella morganii*	·	1/1 (100%)

spp.: species, ESBL: extended-spectrum beta-lactamase, VRE: vancomycin-resistant enterococci, IRAB: imipenem-resistant *Acinetobacter baumannii,* MR-CNS: Methicillin-resistant coagulase-negative *Staphylococci.*

## Discussion

Bactibilia and bacteremia are serious manifestations of acute cholecystitis and are known to be associated with complications, mortality and prognosis [9,10]. The predictive factors for bactibilia have been proposed as advanced age, high body temperature, and increased inflammatory markers such as high serum C-reactive protein [9,11]. However, the association between obesity and bactibilia or bacteremia has not been established.

This study demonstrated that BMI is inversely correlated with bactibilia and bacteremia in patients with acute calculous cholecystitis. According to the study that evaluated the association between BMI and the severity of biliary infections in the United States (US), BMI was not associated with bactibilia. However, BMI was inversely associated with the severity of biliary infections and obesity was postulated as having protective effect on severe infections [12]. From the findings of another study of the Korean population, negative correlation was observed between BMI and the severity of cholecystitis, albeit this was proved only in men [13]. Despite the different study designs and study populations, these studies all indicated that obesity is not associated with severe form or complications of biliary infections. This finding is contrary to the general belief that obese patients are more susceptible to infections. Obesity is also known to be a risk factor for increased morbidity and mortality after infections [14].

Interesting data relevant to this finding is the increased susceptibility or mortality following infection in patients with lower BMI. According to a study of the US population, underweight patients (BMI < 18.5) were associated with increased mortality from non-cancer and non-cardiovascular causes [15]. A Japanese community based study also demonstrated that lower BMI (<18) was associated with mortality of pneumonia, however high BMI (25 >) was postulated as a protective factor [16]. In a study of large Korean cohort, BMI was inversely correlated with mortality from respiratory diseases (tuberculosis, pneumonia, asthma, and chronic obstructive pulmonary disease) [17]. Overall, many ecologic studies indicated that mortality from any cause and BMI shows a J-shaped pattern [17,18]. However, the exact reason or mechanism of increasing infection in patients with lower BMI is yet unclear and it needs to be elucidated from further studies.

Another explanation is the protective effect of obesity against bacterial infections. Obese patients are known to have high plasma lipoprotein levels. Lipoprotein is known to have capacity of binding and neutralizing lipopolysaccharide (LPS) and lipoteichoic acids which are responsible for the activation of *Toll-like receptor* 4 mediated host-immune responses [19,20]. However, general belief is still that patients with obesity are more susceptible to infections [14]. Conflicting results that fit the general belief like impaired lymphocyte proliferation to polyclonal stimulation or pro-inflammatory state of mononuclear cells in obese patients still exist [21,22]. The association between obesity and infection may not be generalized. This might be site-specific. Biliary infections are characterized by ascending form from the duodenum or hematogenous spread from the portal vein, which is called cholangiovenous reflux [12]. According to the studies that evaluated the pathophysiology of biliary bacterial infections, LPS induced inflammatory cytokine activation was the important factor of illness severity [23,24]. It is unclear whether obesity has a positive role in the prevention of biliary infections only. Site-specific studies are needed to confirm this result.

In terms of the hormonal changes of obese patients, adiponectin which is one of the important adipokines is known to reduce proinflammatory cytokines and increase anti-inflammatory cytokines [25]. However, another important adipokine, leptin is known to have the capacity to up-regulate vascular adhesion molecules, and to subsequently induce inflammatory cascade [25]. The combination of these adipokines is associated with metabolic syndrome and the development of cancers, however the association between adipokines and infections (bactibilia or bacteremia) is not well established. Considering the visceral fat that produces various adipokines, studies on the role of visceral fat, including adipokines will give clues about the inverse association between obesity and biliary infections.

Another finding from this study was the bacteriologic profiles of bactibilia and bacteremia. The microbial culture rates of biliary bacteria and bacteremia were not different from those in previous studies (50.4%, 21.6%) [11,12]. *E. coli* and *Klebsiella spp.* were the most prevalent biliary organisms in accordance with the Japanese study [11]. However, the proportion of ESBL and VRE was relatively high which means increased resistance and unresponsiveness to previously used empirical antibiotics. Therefore, interpretation of these results demands caution. Since, many cases were excluded from this study due to the lack of laboratory results of bile and blood cultures, only relatively serious cases could be finally enrolled.

This study has several limitations. It was a retrospective study from single teaching hospital and a small number of patients was enrolled for the statistical analysis. Another limitation is the lacking information about type and number of GB stone and Lipoprotein level was not measured consistently from enrolled patients. However, contrary to the previous studies that assessed the association between obesity and the severity of biliary infections, this study evaluated the direct association between obesity and bactibilia and bacteremia in patients with cholecystitis.

## Conclusions

In conclusion, BMI is inversely correlated with bacteremia or bactibilia, which means obese or overweight patients are less likely to be associated with bacteremia or bactibilia in patients with acute calculous cholecystitis.

## Abbreviations

BMI: Body mass index; OR: Odds ratio; GB: Gallbladder; SD: Standard deviation; HTN: Hypertension; DM: Diabetes mellitus; US: United States; NS: Statistically non-significant; CI: Confidence interval; spp.: Species; LPS: Lipopolysaccharide.

## Competing interests

The authors disclose no financial relationship relevant to this publication.

## Authors’ contributions

CSB participated to study design, data analysis and interpretation, and article drafting. JHY participated to study design, data analysis and interpretation, and gave final approval for publication. YJK participated to data analysis and interpretation. JBK participated to data analysis and interpretation. GHB participated to data analysis and interpretation. KTS participated to data analysis and interpretation. DJK participated to data analysis and interpretation and revising it critically for important intellectual content. All authors read and approved the final manuscript.

## Pre-publication history

The pre-publication history for this paper can be accessed here:

http://www.biomedcentral.com/1471-230X/14/104/prepub
